# Identification of a cuproptosis-related lncRNA signature to predict the prognosis and immune landscape of head and neck squamous cell carcinoma

**DOI:** 10.3389/fonc.2022.983956

**Published:** 2022-12-09

**Authors:** Juntao Huang, Ziqian Xu, Zhechen Yuan, Bing Mei Teh, Chongchang Zhou, Yi Shen

**Affiliations:** ^1^ Department of Otolaryngology Head and Neck Surgery, Ningbo Medical Center Lihuili Hospital, The Affiliated Lihuili Hospital of Ningbo University, Ningbo, Zhejiang, China; ^2^ School of Medicine, Ningbo University, Ningbo, Zhejiang, China; ^3^ Department of Dermatology, Ningbo First Hospital, Zhejiang University, Ningbo, China; ^4^ Department of Ear Nose and Throat, Head and Neck Surgery, Eastern Health, Box Hill, VA, Australia; ^5^ Department of Otolaryngology, Head and Neck Surgery, Monash Health, Clayton, VA, Australia; ^6^ Faculty of Medicine, Nursing and Health Sciences, Monash University, Clayton, VA, Australia

**Keywords:** head and neck squamous cell carcinoma, cuproptosis, long non-coding RNA, prognosis, immunotherapy

## Abstract

**Background:**

Cuproptosis is considered a novel copper-induced cell death model regulated by targeting lipoylated TCA cycle proteins. In this study, we established a novel signature based on cuproptosis-related lncRNAs (crlncRNAs) to predict the prognosis and immune landscape of head and neck squamous cell carcinoma.

**Methods:**

RNA-seq matrix, somatic mutation files, and clinical data were obtained from The Cancer Genome Atlas database. After dividing patients into two sets, a crlncRNA signature was established based on survival related crlncRNAs, which were selected by the univariate Cox analysis and least absolute shrinkage and selection operator Cox regression. To evaluate the model, Kaplan-Meier survival analysis and time-dependent receiver operating characteristic (ROC) were utilized, and a nomogram was established for survival prediction. Immune landscape analysis, drug sensitivity, cluster analysis, tumor mutation burden (TMB) and ceRNA network analysis were conducted subsequently.

**Results:**

A crlncRNA related prognosis signature was finally constructed with 12 crlncRNAs. The areas under the ROC curves (AUCs) were 0.719, 0.705 and 0.693 respectively for 1, 3, and 5-year’s overall survival (OS). Patients in the low-risk group behaved a better prognosis, lower TMB, higher immune function activity and scores. In addition, patients from cluster 2 were more sensitive to chemotherapy and immunotherapy.

**Conclusion:**

In this study, we constructed a novel crlncRNA risk model to predict the survival of HNSCC patients. This reliable and acceptable prognostic signature may guide and promote the progress of novel treatment strategies for HNSCC patients.

## Introduction

As recognized as the sixth leading incidence tumor worldwide, (1–3) the 5-year survival rate of head and neck squamous cell carcinoma (HNSCC) is nearly at 50%. (4, 5) High proliferation, regional lymph node metastasis, and a high recurrence rate contributed to the poor prognosis, which has not significantly improved in the past decade. (6) Although immunotherapy show efficacy to prolong the survival time of cancers, few patients can gain benefit due to the different tumor immune microenvironment. Therefore, it is crucial to explore novel biomarkers and develop novel prognosis signature to predict the prognosis of HNSCC patients and provide the precise and individual treatment.

Cuproptosis, which is regulated by copper ions, is considered a novel copper-induced cell death model determined in recent research. (7) As Tsvetkov et al. reported, this cooper-dependent biological process was regulated by targeting lipoylated components of the tricarboxylic acid (TCA) cycle. (8) Core cuproptosis-related genes (CRGs) play important roles in the lipoic acid pathway and are protein targets of lipoylation. Among them, FDX1 and protein lipoylation are considered the key regulators of copper ionophore-induced cell death. (8) According to previous studies, cell death participates in the regulation of cell biological processes and is closely associated with tumor cell proliferation, migration and invasion (3, 9), which suggested cuproptosis may behave potential possibility to influence prognosis and tumor immune microenvironment. Previous studies have explored the correlation of different cell death models and tumors; however, as a novel cell death model, there is a lack of studies investigating the relationship between cuproptosis and HNSCC. This novel biological progress performs great potential to predict the prognosis and guide the immunotherapy in HNSCC patients.

Long non-coding RNAs (lncRNAs) are gradually considered as important factors in the biological progression of HNSCC. (3, 10, 11) Referring to previous studies, lncRNAs can promote reprogramming in cancers; (12) however, the correlation between lncRNAs and cuproptosis in HNSCC is also required to be further explored. Hence, in this study, we divided HNSCC patients into low- and high-risk groups based on cuproptosis-related lncRNAs (crlncRNAs) and subsequently constructed a novel prognostic risk model to predict the prognosis of HNSCC and the immunotherapeutic efficacy.

## Methods and materials

### Downloading the HNSCC datasets and clinical data

The HNSCC gene expression dataset with detailed clinical information was obtained from The Cancer Genome Atlas (TCGA) database (last assessed on April 12, 2022). This dataset was consisted of 504 HNSCC samples and 44 normal samples. After downloading transcriptome data as fragments per kilobase million (FPKM), TCGA-HNSC associated clinical information were also summarized, including age, gender, grade, clinical tumor stage, T stage, N stage, M stage, overall survival (OS) status, and survival value. Patients with short OS values (less than 30 days) or missing OS values were excluded to decrease the potential bias. Subsequently, patients in the entire dataset were divided into a training set and a test set randomly at a ratio of 1:1 for further analysis.

### Obtaining the expression of crlncRNAs in HNSCC

A total of 19 CRGs, including FDX1, LIPT1, LIPT2, LIAS, DLD, DLAT, PDHA1, PDHB, MTF1, GLS, CDKN2A, ATP7A, ATP7B, DBT, GCSH, DLST, NFE2L2, NLRP3 and SLC31A1, were extracted and obtained by screening previous studies. (8, 13, 14) Subsequently, after preparing 19 CRGs and all lncRNAs expression matrix of TCGA-HNSC dataset respectively, the Pearson correlation analysis was conducted to select the crlncRNAs by the use of the “limma” R packages. Based on the criteria of |correlation coefficients| > 0.4 and P< 0.001 with 19 CRGs, the crlncRNAs of the TCGA-HNSC cohort were distinguished and selected by Pearson correlation analysis, considering as associated with cuproptosis (15–17).

### Construction of the crlncRNA prognostic model

The survival related crlncRNAs in the training set were assessed and selected by univariate Cox proportional hazards regression analysis with the criteria of P value less than 0.05. After identifying the eligible survival related crlncRNAs, least absolute shrinkage and selection operator (Lasso) Cox regression with P value of 0.05 and performed with 10-fold cross-validation to identify the crlncRNAs for the risk model. The analysis was performed for 1,000 cycles to prevent overfitting and the multivariate Cox regression analysis was subsequently to further selected the eligible crlncRNAs and calculated their coefficients in the novel risk models. Based on above results, patients were assessed by the risk scores according to the following formula: risk score= Σ coefficient of (crlncRNAi) * (crlncRNA^i^) expression. Furthermore, the HNSCC patients of the entire TCGA-HNSC set were grouped as two groups (low-risk and high-risk) in according with the median value of risk scores. Besides, the differences in OS between these two groups were compared using Kaplan-Meier (KM) survival analysis.

Respectively, the survival receiver operating characteristic (ROC) curves of 1-, 3-, and 5-year and their areas under the curves (AUCs) were utilized to evaluate the effects of prediction, comparing with other clinical features of age, gender, grade and clinical stages. The C-index was also used to test the predictive efficacy of the predict signature. Moreover, based on the results of univariate and multivariate Cox regression analysis, a survival prediction nomogram was established based on the risk groups and survival-related clinical information. Besides, the degree of consistency about calibration plots were certified by the use of the Hosmer-Lemeshow goodness of fit test to assess reliability of the nomogram.

### Signature enrichment analysis, protein-protein interaction network, gene ontology, Kyoto encyclopedia of genes and genomes pathway and gene set enrichment analysis

The cancer-related signature enrichment analysis was conducted to explore the signature crlncRNAs enriched situation within the relevant category by the use of LncSEA database (http://www.licpathway.net/LncSEA/). (18) Similarly, to identify the potential interaction of differentially expressed genes (DEGs) between the two risk groups, the Search Tool for the Retrieval of Interacting Genes/Proteins (STRING) (http://string-db.org/) database was utilized to explore the protein-protein interactions (PPIs). DEGs were differentiated in accordance with the criteria of |logFC| ≥ 0.585 and false discovery rate (FDR)< 0.05. Then, gene ontology (GO) analysis and Kyoto Encyclopedia of Genes and Genomes (KEGG) pathway analysis, were performed as functional enrichment analysis to elucidate the underlying mechanism of DEGs. With the utilization of “clusterProfiler” and “bioconductor” R packages, the related assessments of biological processes (BPs), cellular components (CCs), molecular functions (MFs), and key signaling pathways were performed. The significantly enriched GO and KEGG terms were considered and selected, while p values and q-values were both less than 0.05.

Gene set enrichment (GSEA) analysis was also conducted by using GSEA software based on the assisted gene set (kegg.v7.5.1.symbols.gmt) The identified pathways in the two risk groups were identified and visualized in accordance with the criteria of FDR< 0.05.

### Correlation analysis between risk models and tumor mutation burden

With the utilization of the “maftool” R package, the top 15 most commonly mutation genes in HNSCC patients of the low- and high-risk groups were compared and visualized *via* waterfall plots. Comparison of risk scores in the wild type (WT) and mutation type (MT) of the topmost mutated gene was also performed. Moreover, to further compare the tumor mutation burden (TMB) of two risk groups. The correlation between two risk groups and TMB frequency was displayed and compared through the Wilcoxon signed-rank test.

### Comparison of immune landscape between the low- and high-risk groups

Subsequently, the immune cell infiltration statuses were analyzed *via* the tool CIBERSORT and summarized as a boxplot diagram. Similarly, the differences in infiltrated immune cells and immune functions between the low- and high-risk groups were compared *via* the single sample GSEA (ssGSEA) method. Moreover, the tumor microenvironment (TME) scores for each sample were calculated with the use of the “estimate” package of the ESTIMATE algorithm. Besides, the differences in stromal scores, immune scores, and ESTIMATE scores were compared between the two risk groups and displayed in boxplots.

### Assessment of clinical treatment based on the risk scores

The effectiveness of four chemotherapy drugs commonly utilized in HNSCC patients, including cisplatin, paclitaxel, docetaxel, and gemcitabine, was analyzed and compared. Values of half-maximal inhibitory concentration (IC50) for these four chemotherapeutic drugs of each HNSCC patient were utilized to assess and compare the chemotherapy response between the two risk groups using the “pRRophetic” package. Patients with lower IC50 values in groups were considered to have better drug sensitivity to these four chemotherapeutic agents while the statistical P value was less than 0.05, as assessed by the Wilcoxon signed-rank test (19).

Similarly, the relationships between the risk groups and expression of immune checkpoints (e.g., PD-1, CTLA4 and PD-L1) were calculated and compared by the use of Wilcoxon signed-rank test analysis. In addition, the potential effectiveness of the immunotherapy response was also evaluated and compared based on the immunophenoscore (IPS) of each HNSCC sample from The Cancer Immunome Atlas (TCIA) database (https://tcia.at/home).

### Cluster analysis based on prognostic crlncRNAs

To explore the potential molecular subtypes with different immunotherapy response, cluster analysis was performed with prognostic crlncRNAs, which were used to establish the risk model and considered strongly associated with survival. Based on expression of these prognostic crlncRNAs, patients were then divided into different subtypes using the “ConsensusClusterPlus” package. Subsequently, K-M survival analysis were applied to compared the differences of OS in clusters. Similarly, principal component analysis (PCA), as well as t-distributed stochastic neighbor embedding (t-SNE) analysis, were also performed to evaluate the clusters. Furthermore, immune-related analysis, including immune cells infiltration, TME scores and immune function, and assessment of immunotherapy and chemotherapy treatment were conducted in clusters for further exploration.

### Establishment of lncRNA-miRNA-mRNA competing endogenous RNA

In order to further explore the targeted and intersection relationship crlncRNAs and (CRGs), the correlation analysis between prognostic crlncRNAs and 19 CRGs was analyzed by the use of the “limma” and “heatmap” R packages. Subsequently, we used the targeted network to explain the potential correlation between crlncRNAs and CRGs by their common targeted miRNAs. The differentially expressed CRGs and micro RNAs (DEMs) between HNSCC samples and normal samples of the TCGA dataset were identified with the criteria of |logFC| ≥ 1 and FDR< 0.05 for establishing a competing endogenous RNA (ceRNA) network of prognostic crlncRNA, DEMs, and CRGs. The DRGs-related miRNAs were predicted by the Encyclopedia of RNA Interactomes (ENCORI) database (https://starbase.sysu.edu.cn/) and were compared with DEMs to select the intersected miRNAs for further analysis. Then, the intersected lncRNAs between the predicted targeted lncRNAs and prognostic crlncRNAs were also screened to construct the network *via* Cytoscape version 3.6.2 software. Moreover, patients of the entire cohort were respectively divided into low-expression and high-expression groups based on elements of ceRNA networks, and survival analysis was performed to independently explore their influence on prognosis. In addition, the correlation between the single CRGs, crlncRNAs of the ceRNA network and immune cells was assessed based on the CIBERSORT method.

## Results

### Study design and expression of crlncRNAs in HNSCC patients

**Figure 1:**
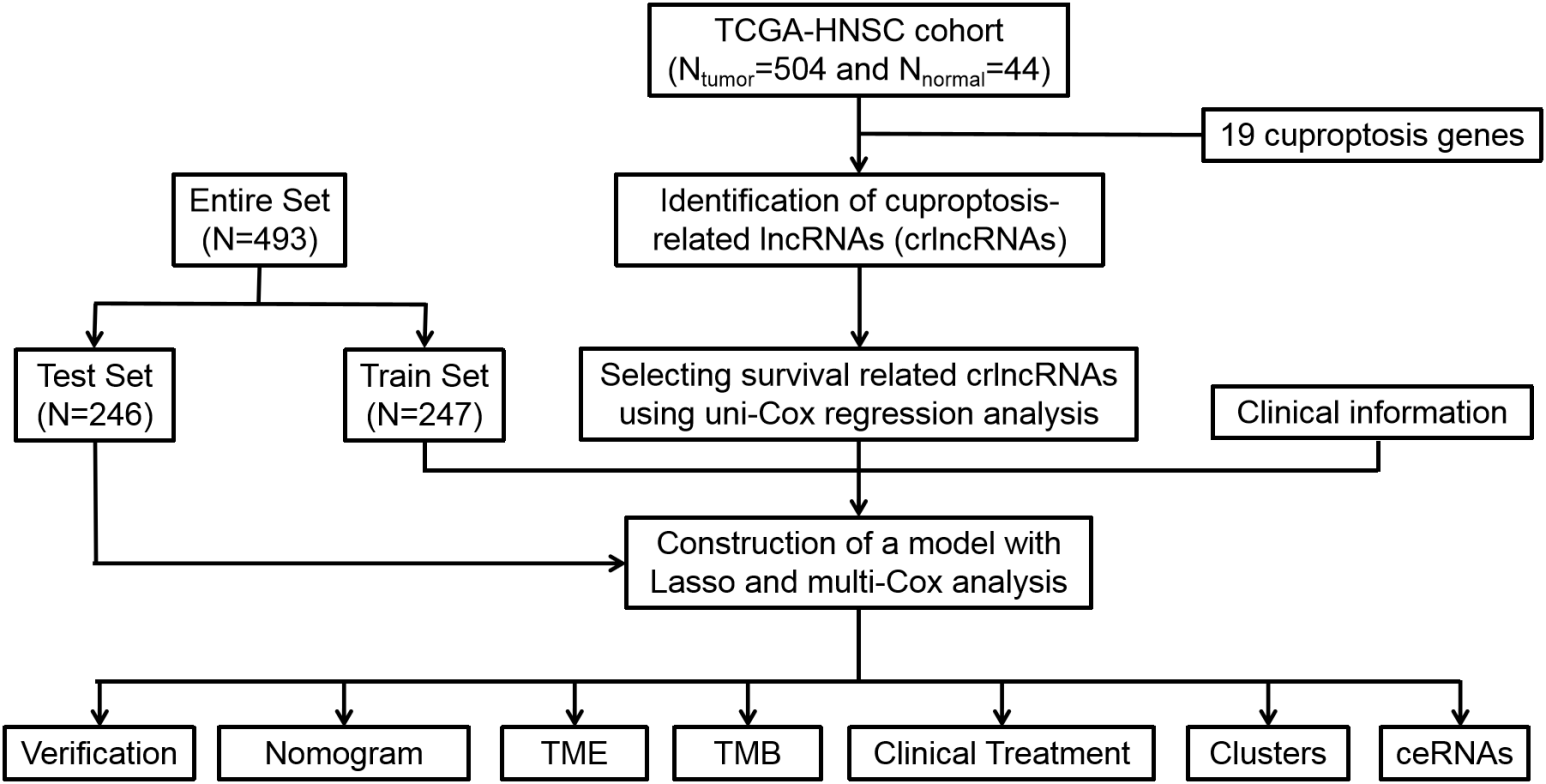
Flow diagram of study design. Lasso: least absolute shrinkage and selection operator; TME, tumor microenvironment; TMB, tumor mutation burden, ceRNA, competing endogenous RNA.

The study design of this analysis is shown as a flowchart in **Figure 1**. Patients of TCGA-HNSC dataset were subsequently and randomly divided into the training cohort and test cohort at a ratio of 1:1. Based on the selection criteria of correlation coefficient > 0.4 and P< 0.001, 783 crlncRNAs were identified.

### Construction and verification of the crlncRNA risk model

**Figure 2:**
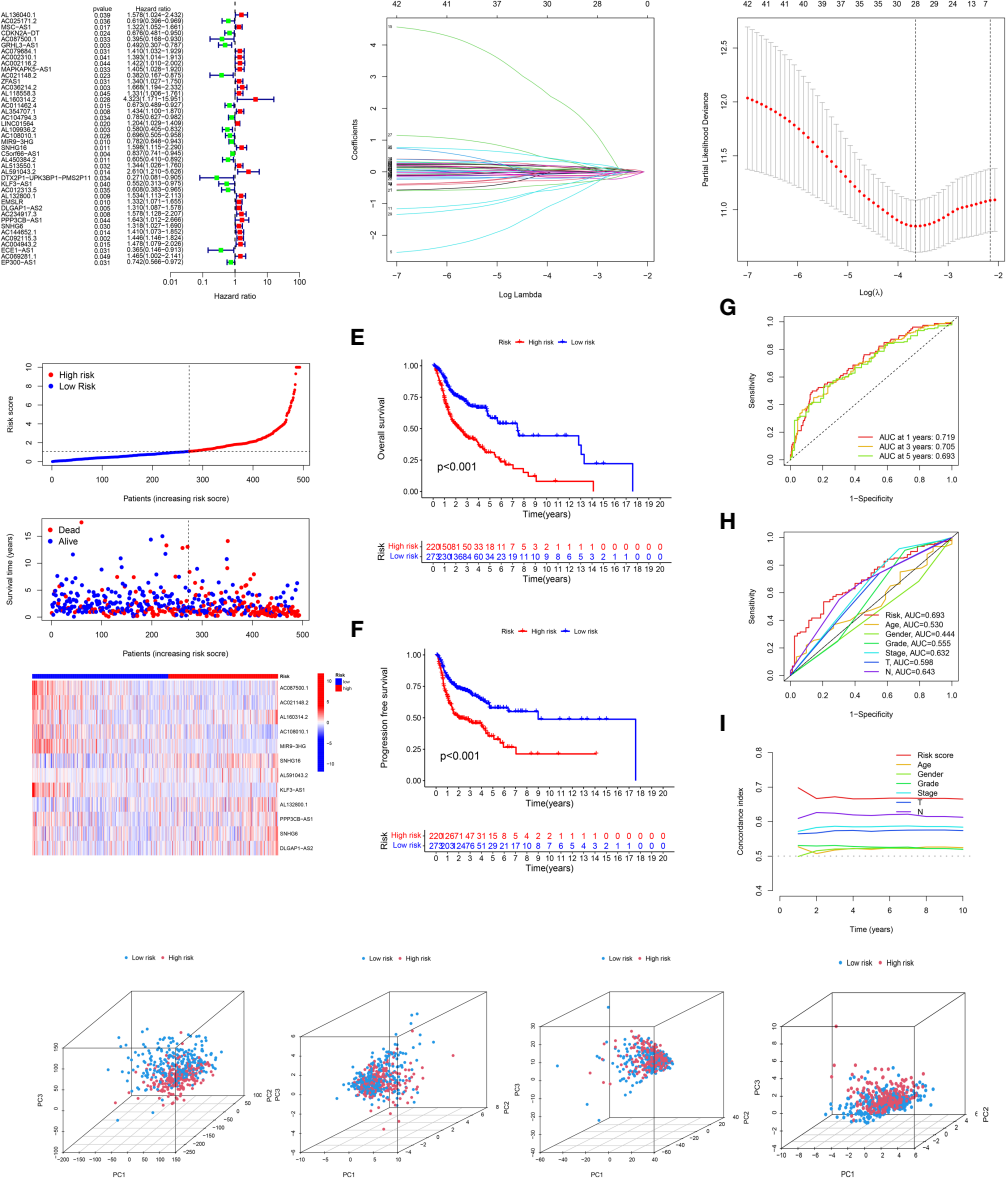
Expression of cuproptosis-related lncRNAs (crlncRNAs) in TCGA-HNSCC dataset and development of a crlncRNAs prognosis signature. **(A)**, Forest plot showing the prognostic value of crlncRNAs based on the univariate Cox proportional hazards regression analysis (P < 0.05); **(B)**, Diagram for least absolute shrinkage and selection operator (Lasso) expression coefficients; **(C)**, Cross-validation plot for the penalty term of Lasso analysis; **(D)**, Exhibition, survival time and survival status and crlncRNA expression heatmap between low-risk and high-risk groups. **(E)**, K-M survival curves of OS in entire TCGA-HNSC dataset; **(F)**, K-M survival curves of progression free survival in TCGA-HNSC dataset; **(G)**, Receiver operating characteristic (ROC) curves of the risk model; **(H)**, Comparison of areas under the curves (AUC) values of 5-year’s ROC curves among risk model and clinical features; **(I)**, C-index of risk score and clinical characteristics; **(J)**, Principal component analysis to show the distinguish of all gene, cuproptosis-related gene, cuproptosis-related lncRNAs and risk model lncRNAs.

Concerning the results of univariate Cox proportional hazards regression analysis (**Figure 2A**), a total of 42 crlncRNAs related to survival in the training cohort were selected. Among them, 12 were considered as prognostic factors which may lead to worse prognosis for HNSCC patients (hazard ratio, HR>1); nevertheless, the remaining crlncRNAs decreased the risks of patients. Subsequently, multivariate Cox regression and Lasso Cox regression analyses were utilized to construct the prognostic model based on these crlncRNAs. (**Figures 2B, C**) HNSCC patients were then divided into low- and high-risk groups according to the median risk score. (**Figure 2D**) The risk scores of HNSCC patients were calculated with the following formula: Risk score= AC087500.1 × (-2.11301404241346) + AC021148.2 × (-0.857608193486481) + AL160314.2 × 3.51329203110076 + AC108010.1 × (-0.718805974335349) + MIR9-3HG × (-0.298522856210143) + SNHG16 × 0.551257405027881 + AL591043.2 × 0.749790597996073 + KLF3-AS1 × (-0.999098398001405) + AL132800.1 × 0.387234548382698 + PPP3CB-AS1 × 0.641016534829951 + SNHG6 × 0.359103642883439 + DLGAP1-AS2 × 0.201117298899406.

**Figure 3:**
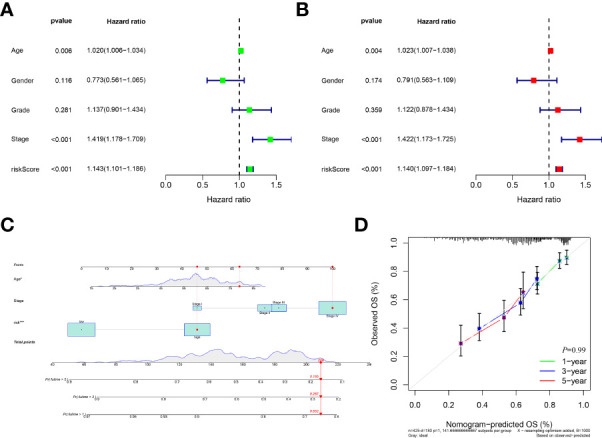
Development a nomogram. **(A)**, Forest of uni-Cox analyses among clinical features and risk scores. **(B)**, Forest of multi-Cox among clinical features and risk scores. **(C)**, Establishment of nomogram to predict the 1-, 3-, and 5-year’s prognosis based on ages, stages and risk scores; **(D)**, Calibration curves plot and Hosmer-Lemeshow goodness of fit test indicating high degree of consistency and reliability of the nomogram.

According to the risk score system, patients were compared in terms of survival status and time, suggesting that patients in the low-risk group behave better prognoses in any clinical subgroups. (**Figures 2E, F** and **Supplementary Figure S1**) Moreover, the AUC values of the 1-year, 3-year and 5-year survival ROCs were respectively of 0.719, 0.705 and 0.693. Comparing the 5-year AUC values with other features, the risk model performed better in predicting efficacy than other clinical factors, including age (0.530), sex (0.444), grade (0.555), clinical stage (0.632), T stage (0.598) and N stage (0.643). (**Figure 2H**) Similar results were also constructed by the analysis of the C-index. (**Figure 2I**) The PCA determined that the two risk groups could be distinguished clearly. (**Figure 2J**) In addition, according to the results of uni-Cox and multi-Cox analyses, the risk score (HR_uni-Cox_=1.143 and HR_multi-Cox_=1.140), age and stage were considered three prognostic parameters influencing the final prognosis. (**Figures 3A, B**) Based on these independent factors, a nomogram was established to predict HNSCC patient survival probability. (**Figure 3C**) In terms of the 1-, 3- and 5-year survival calibration plots and the Hosmer-Lemeshow goodness of fit test (P=0.99), the high degree of consistency certified the reliability of the nomogram (**Figure 3D**).

### LncSEA, GO, KEGG, PPI and GSEA analyses

The enriched status of 12 prognostic crlncRNAs in relevant category was shown in **Supplementary File**. As indicated by the LncSEA database, these crlncRNAs were considered strongly associated with survival of HNSCC (P<0.05) as well as other biological category (e.g, transcription factor). In total, 1492 genes were differentially expressed between the low- and high-risk groups. As shown in the circle plot of GO analysis, these DEGs were mostly enriched in immune-related BPs (e.g., B-cell activation), CCs (e.g., immunoglobulin complex), and MFs (e.g., antigen binding) (**Supplementary Figure S2A**). The results of KEGG pathway analysis suggested that these DEGs were mostly associated with cytokine-cytokine receptor interactions, chemokine signaling pathways, cell adhesion molecules and so forth (**Supplementary Figure S2B**). The PPI network was established and is shown in **Supplementary Figure S2C**.

GSEA was also performed to explore the differences between the two risk groups of the entire set. With the criteria of FDR<0.05, nine pathways were more enriched in the high-risk group, and three pathways were more activated in the low-risk group. All |NESs| values of pathways were > 1.5 and were considered highly correlated with tumor invasion and immunity (**Supplementary Figure S2D**).

### Correlation analysis between risk models and TMB

**Figure 4:**
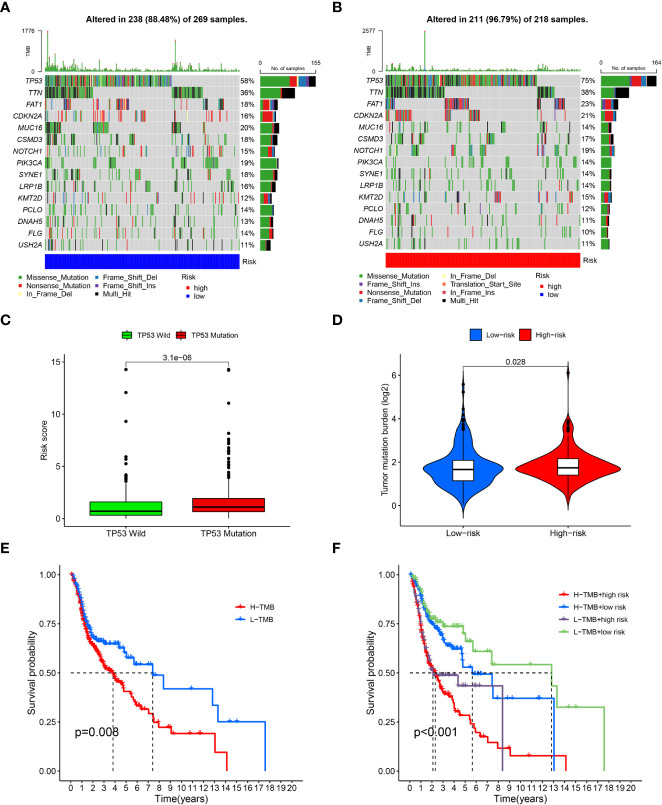
Tumor mutation burden analysis. **(A)**, Waterfall plots of topmost 15 mutated genes in the low-risk group; **(B)**, Waterfall plots of mutation in the high-risk group; **(C)**, Correlation analysis of TP53 wild type, TP53 mutation type and risk scores; **(D)**, Comparison of tumor mutation burden (TMB) between the low-risk and high-risk groups; **(E)**, survival analysis between high and low TMB cohorts; **(F)**, survival analysis for patients combined with TMB and risk scores.

After dividing the somatic mutation information into two groups based on the risk scores, the differences of the 15 topmost mutated genes between the low- and high were shown in **Figures 4A, B**. As the waterfall plots suggested, the topmost commonly mutated genes were TP53. As shown in **Figure 4C**, TP53 MT had higher risk scores than TP53 WT. Furthermore, while comparing the frequency of TMB between the two risk groups, the high-risk group exhibited significantly higher TMB frequencies than the low-risk group. (**Figure 4D**) Specifically, based on the survival analysis, patients with higher TMB had worse OS than those with lower TMB (**Figures 4E, F**).

### Immune landscape analysis

**Figure 5:**
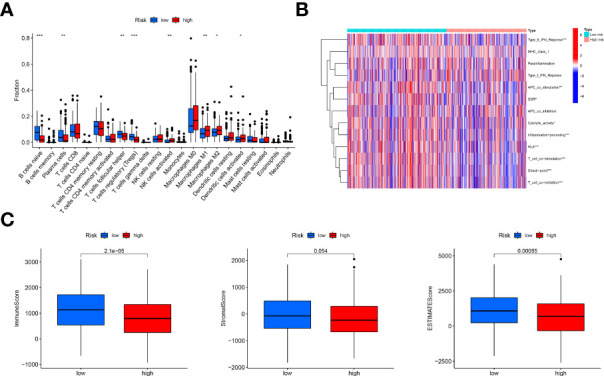
Immune landscape analysis. **(A)**, Comparison of immune cells infiltration analysis between the low-risk and high-risk groups based on CIBERSORT, suggesting more immune cells infiltrated in the low-risk group; **(B)**, Heatmap of immune function comparison between two risk groups using the ssGSEA analysis, indicating more immune function enriched in the low-risk groups; **(C)**, Comparison of immune scores, stromal score and ESTIMATE score based on the method of estimate, showing high immune-related scores in the low-risk groups. "*, ** and ***" indicated P value was less than 0.05, 0.01 and 0.001 respectively.

According to the results of CIBERSORT, naive B cells, plasma cells, follicular helper T cells and T regulatory cells (Tregs) were negatively associated with the risk scores; nevertheless, the high-risk group exhibited increased infiltration of activated NK cells, M1 macrophages, M2 macrophages and activated dendritic cells infiltrated. (**Figure 5A**) As for regarding immune function, the low-risk group had more associated immune functions, including the type II IFN response, cytolytic activity, CCR, inflammation promotion, HLA, T-cell costimulation, checkpoint and T-cell coinhibition. (**Figure 5B**) Furthermore, the low-risk group had a higher immune score and ESTIMATE score; however, there were no differences in stromal score between the two risk groups (**Figure 5C**).

### Assessment of chemotherapy and immunotherapy

**Figure 6:**
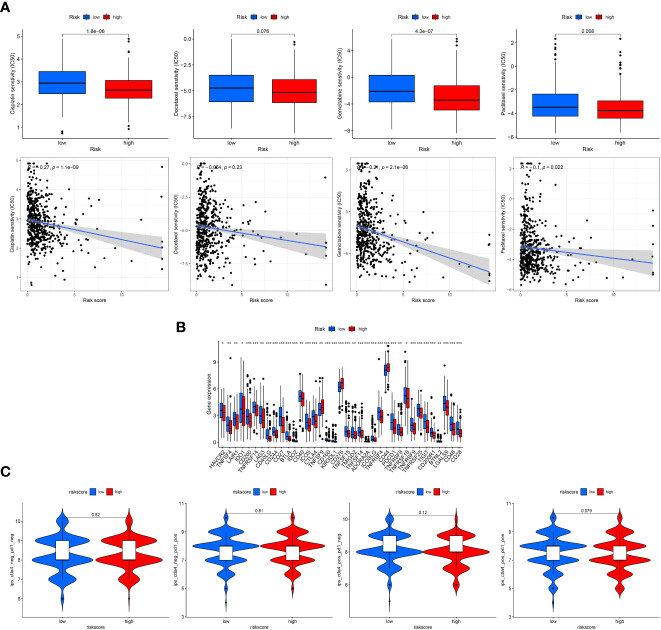
Clinical treatment assessment. **(A)**, Drug sensitivity comparison of cisplatin, paclitaxel, docetaxel, and gemcitabine between the low- and high-risk groups and correlation with risk scores. IC50, half-maximal inhibitory concentration. **(B)**, Comparative expression of immune checkpoint genes in risk groups. **(C)**, Comparison of immunophenoscore (IPS) in the two groups based on TCIA database to predict the immunotherapeutic response of PD-1 and CTLA-4 inhibitors. "*, ** and ***" indicated P value was less than 0.05, 0.01 and 0.001 respectively.

With the use of the “pRRophetic” R package, the drug sensitivity of four chemotherapy agents were compared and evaluated between the two risk groups with the IC50 values. As reflected in **Figure 6A**, the IC50 values of these four chemotherapy drugs had a negative correlation with risk scores, and patients in the high-risk scores exhibited higher drug sensitivity to cisplatin, gemcitabine and paclitaxel than the low-risk groups.

In addition, patients in the low-risk group displayed more checkpoint activation than those with high-risk scores (**Figure 6B**), including PD-1 and CTLA4. However, the violin charts based on IPS also confirmed that patients displayed similar immunotherapeutic responses to PD-1 inhibitor therapy alone, CTLA4 inhibitor therapy alone or the combination of PD-1 and CTLA4 (**Figure 6C**).

### Cluster analysis based on prognostic crlncRNAs

**Figure 7:**
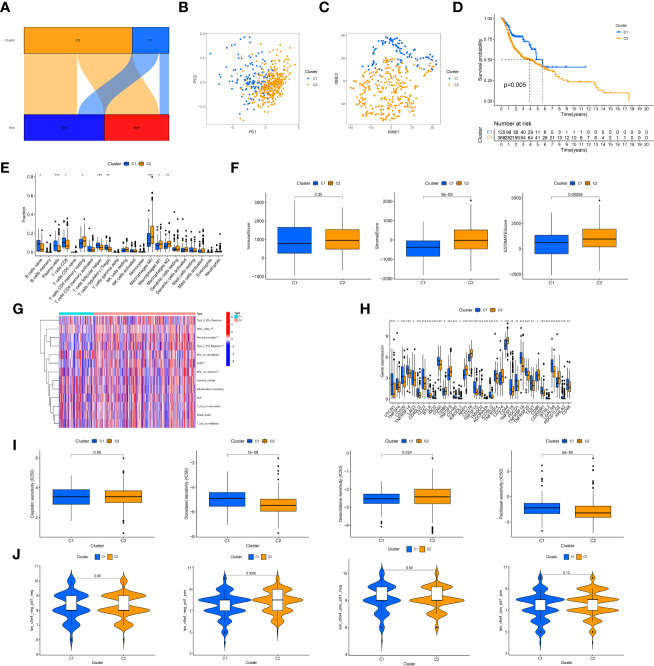
Cluster analysis. **(A)**, Sankey diagram of relationship between clusters (bright blue: cluster 1; yellow: cluster 2) and risk groups (dark blue group: low-risk group; red: high-risk group). The axes suggested most of patients in the high-risk group were regrouped into cluster 2. **(B)**, The principal component analysis of clusters to reflect the distinguish of genes by two clusters; **(C)**, The t-distributed stochastic neighbor embedding analysis of clusters to reflect the distinguish of genes by two clusters; **(D)**, K-M survival curves of OS in clusters; **(E)**, Immune cells infiltration analysis in clusters; **(F)**, Tumor microenvironment scores of two clusters; **(G)**, Comparison of immune functions; **(H)**, Different expression of immune checkpoints in clusters; **(I)**, Comparison of IC50 value of cisplatin, paclitaxel, docetaxel, and gemcitabine in clusters; **(J)**, Comparison of IPS in clusters. "*, ** and ***" indicated P value was less than 0.05, 0.01 and 0.001 respectively.

Based on the expression of 12 prognostic crlncRNAs, patients were then divided into two subgroups with the use of the “ConsensusClusterPlus” R package. (**Figure 7A**) PCA (**Figure 7B**) and t-SNE (**Figure 7C**) suggested that the two clusters behave clear differences. The K-M survival analysis indicated that patients in cluster 2 had worse OS than those in cluster 1. (**Figure 7D**) According to the analysis of immune infiltration by CIBERSORT, cluster 2 was enriched in immune cells, including resting memory CD4 T cells, M0 macrophages, M1 macrophages, and M2 macrophages. (**Figure 7E**) The results of the TME shown in the boxplots also suggested that cluster 2 had a higher stromal score and ESTIMATE score than cluster 1. (**Figure 7F**) Similarly, patients in cluster 2 were more active in immune functions (e.g., parainflammation) than those in cluster 1 based on ssGSEA. (**Figure 7G**) For the assessment of immune checkpoints, patients in cluster 2 displayed higher expression of CD274, CD276 and PDCD1LG2. (**Figure 7H**) Furthermore, patients in cluster 2 were more sensitive to docetaxel and paclitaxel; nevertheless, cluster 1 had a lower IC50 value of gemcitabine. (**Figure 7I**) The IPS results showed that cluster 2 showed a better immunotherapeutic response to PD-1 inhibitor therapy (**Figure 7J**).

### Construction of the ceRNA network

**Figure 8:**
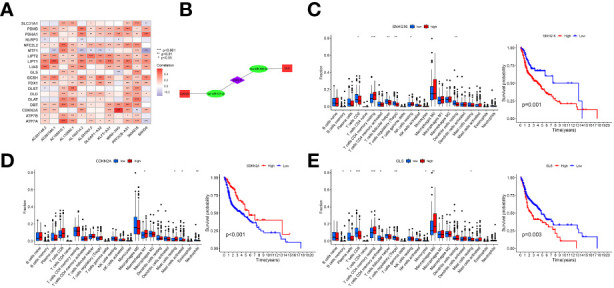
Correlation of prognostic crlncRNAs and CRGs. **(A)**, Heatmap indicating the correlation between crlncRNAs and CRGs; **(B)**, crlncRNA-miRNA-mRNA ceRNA network suggesting potential targeting intersection. **(C, E)**, Immune cells infiltration and K-M survival curves of OS based on the expression of SNHG16 **(C)**, CDKN2A **(D)**, and GLS **(E)**.

The correlation between CRGs and prognostic crlncRNAs is shown in **Figure 8A**. Furthermore, to explore the potential correlation between prognostic crlncRNAs (establishing the risk model) and CRGs, we established the ceRNA network to predict the targeting intersection with the use of their common targeted miRNAs. With the criteria of |logFC| ≥ 1 and FDR< 0.05, two CRGs (CDKN2A and GLS) and 86 miRNAs were considered differentially expressed between HNSCC and normal samples. After selecting the intersection between DEMs and predictive results, SNHG16 was considered to be a related prognostic lncRNA with the ENCORI database, and the ceRNA network was established and is shown in **Figure 8B**. Moreover, the survival analysis indicated that HNSCC patients with lower SNHG16 expression (as well as GLS) or higher CDKN2A expression displayed better OS. The CIBERSORT analysis also indicated immune cell infiltration in HNSCC samples considering the expression of SNHG16, CDKN2A and GLS (**Figures 8C, E**).

## Discussion

For patients diagnosed with HNSCC, the 5-year survival rates were extremely poor according to previous studies (4, 5). It is crucial and necessary to predict prognosis and guide treatment. However, there is a lack of reliable biomarkers or prediction models to forecast HNSCC progression. (20, 21) Cuproptosis is reportedly involved in regulating cell death by copper ions. (8) This cell death model can be considered a novel method to influence tumor progression and suppression, as well as novel therapeutic targets for HNSCC patients. However, the relationship between CRGs and HNSCC remains unknown, and there is lack of related cuproptosis predict models for HNSCC patients. Given this, we conducted this analysis to constructed a novel crlncRNA signature to predict the prognosis and immune landscape of HNSCC.

According to our analysis, this prognostic risk model divided HNSCC patients into low- and high-risk groups. For patients with different clinical features, patients in the low-risk group had better OS than those in the high-risk group, suggesting that the risk score was negatively correlated with OS. The 5-year AUC value of the risk model was higher than that of other clinical factors, indicating better prognostic effects in HNSCC patients. Additionally, as independent risk indicators, the nomogram was established based on the risk score and two other clinical characteristics (age and stage). The calibration plots of 1-, 3- and 5-year determined a high degree of consistency. Above all, these results indicated that this crlncRNA signature demonstrated high robustness and efficacy in predicting the prognosis of HNSCC.

Among these 12 prognostic crlncRNAs, the lncRNA of SNHG16 was upregulated in laryngeal squamous cell carcinoma tissues (LSCC) by regulating the miR-140-5p/NFAT5/Wnt/β-catenin pathway axis, which may possibly provide a novel method for HNSCC treatment. (22) Li et al. also determined that the lncRNAs of SNHG16 can enhance the progression and carcinogenesis in oral squamous cell carcinoma. SNHG6 showed significant differential expression both *in vitro* qRT-PCR and in silico analysis (23). In addition, AC108010.1 (24) and MIR9-3HG (25) were identified as ferroptosis-related lncRNAs to establish a prognostic signature. Moreover, CDKN2A is considered a common mutation of the tumor suppressor and checkpoint mediator in HPV-negative HNSCC (26). GLS can facilitate HNSCC cell proliferation, migration, invasion and glutamine catabolism by regulating hsa-circ-0000003 *via* the miR-330-3p/GLS pathway (27). Furthermore, hsa-miR-124-3p (28) and hsa-miR-488-3p (29) were also determined to play crucial roles in HNSCC and other tumors according to previous studies. Therefore, the ceRNA network indicated and predicted the potential relationship of crlncRNAs, miRNAs and CRGs in metabolizing HNSCC tumor cell biological processes.

Previous studies have indicated that TMB may play important roles in immune cell infiltration and influence clinical effectiveness of immunotherapy (30, 31). According to our analysis, patients with high risk scores had a higher TMB than those with low risk scores. The survival comparison between the two risk groups indicated that HNSCC patients with a higher TMB had a worse prognosis, which coincided with Zhang et al.’s study (32). Moreover, as reported in previous studies, TMB is associated with CD4+ Tell and B-cell infiltration status, which were also determined in our analysis (32).

Importantly, the TME plays important roles in tumor immunotherapy. (33, 34) Based on the methods of CIBERSORT and ESTIMATE, HNSCC patients in the low-risk group were more associated with infiltration of plasma cells and follicular helper T-cell, as well as high immune and ESTIMATE scores. Similarly, ssGSEA suggested that patients with low-risk scores had more active immune functions than those with high risk scores. In addition, patients in the low-risk group had higher checkpoint gene expression (35). Given these findings, patients with lower risks may have a better immunotherapy response. However, there were no significant differences in PD-1 and CTLA-4 expression between the two groups. The TCIA analysis also supported the results of similar immunotherapeutic effects of PD-1 or CTLA-4 in the two groups. Moreover, the risk scores show significant negative correlations with the IC50 values of cisplatin, gemcitabine and paclitaxel, indicating that patients with high risks have a better chemotherapeutic response.

Subsequently, after dividing the TCGA cohort into two clusters, patients in cluster 2, which mostly consisted of high-risk group patients, had worse OS and better immunotherapy and chemotherapy responses. These novel subtypes are considered clinically significant to guide the development of individualized and precise treatment in clinics.

There are also several limitations in our study. First, there is a lack of lncRNA expression matrix of HNSCC and associated clinical information from external databases, which may consider as external cohorts for test. Although the prognostic effects of the risk signature are reliable, prospective studies are needed to test the results of our bioinformatics. Similarly, as a novel cell death model, the mechanism and pathway of cuproptosis requires more experimental studies, therefore, based on currently few researches, it is difficult to conduct the analysis of pathways associated with cuproptosis in this manuscript now. Clinical trials with large samples are also required to investigate the effectiveness of immunotherapy and chemotherapy with signatures.

## Conclusion

In this study, we constructed a novel crlncRNA risk model to predict the survival of HNSCC patients. This reliable and acceptable prognostic signature may guide and promote the progress of novel treatment strategies for HNSCC patients.

## Data availability statement

The original contributions presented in the study are included in the article/**Supplementary Material**. Further inquiries can be directed to the corresponding authors.

## Author contributions

All persons designated as the authors have participated sufficiently in the work to take public responsibility for the content of the manuscript.

## Supplementary material

Supplementary Figure 1 Subgroup K-M survival analysis of entire dataset based on different clinical characters.

Supplementary Figure 2 S2A, Circle plot and bar plot about Gene Ontology analysis; S2B, Circle plot and bubble plot Kyoto Encyclopedia of Genes and Genomes Pathway analysis; S2C, Protein-protein interaction network; S2D; Gene Set Enrichment Analysis.
