# Supplementary figures and images for: Identification of a cuproptosis-related lncRNA signature to predict the prognosis and immune landscape of head and neck squamous cell carcinoma
Source: Front Oncol. 2022 Dec 9;12:983956. doi: 10.3389/fonc.2022.983956 (PMC9780454; doi:10.3389/fonc.2022.983956)

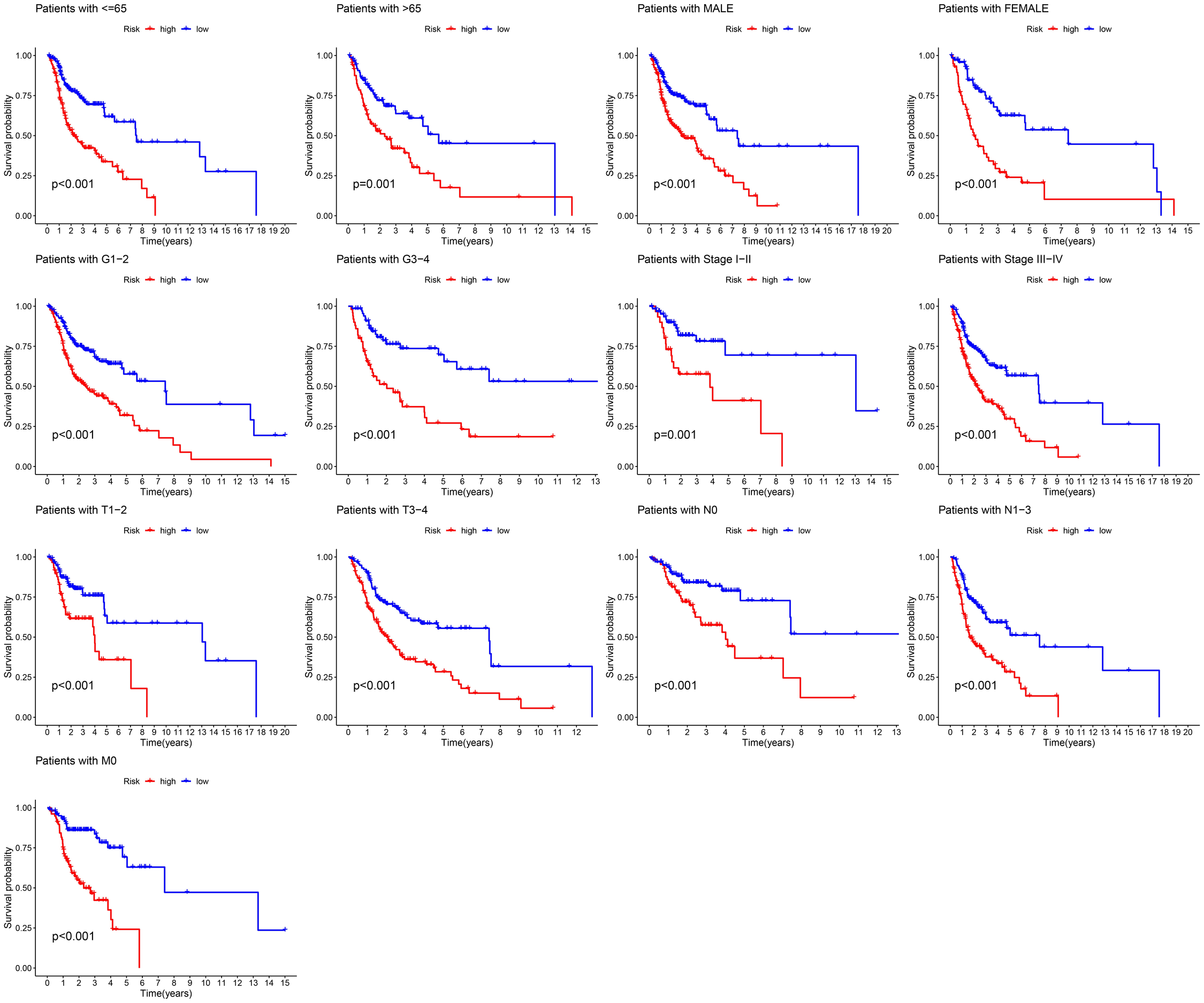

Supplement: Supplementary Figure 1 — Subgroup K-M survival analysis of entire dataset based on different clinical characters. [file Image_1.tif]

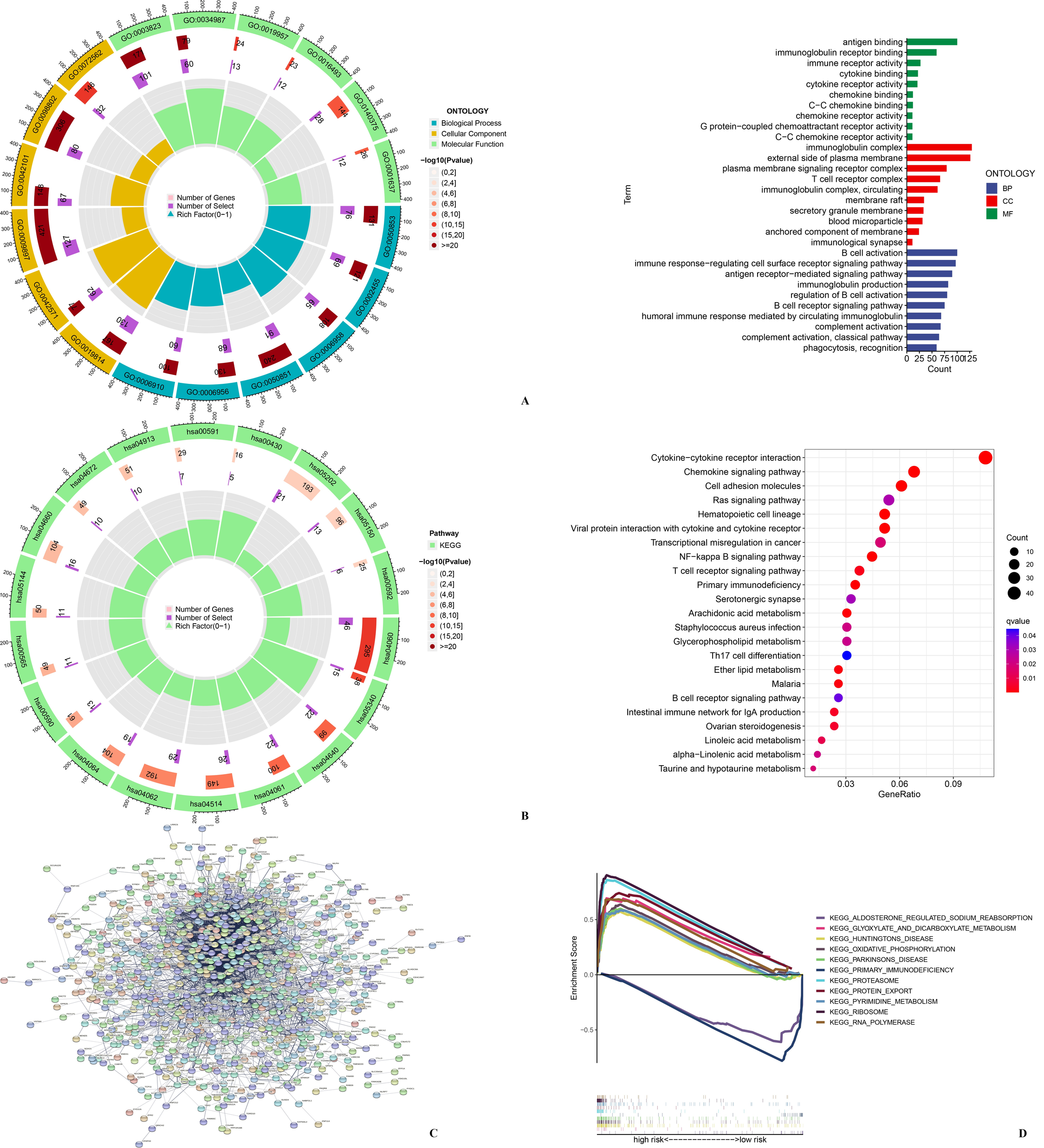

Supplement: Supplementary Figure 2 — S2A, Circle plot and bar plot about Gene Ontology analysis; S2B, Circle plot and bubble plot Kyoto Encyclopedia of Genes and Genomes Pathway analysis; S2C, Protein-protein interaction network; S2D; Gene Set Enrichment Analysis. [file Image_2.tif]
